# Supplementary figures and images for: Comprehensive Phosphoproteomic Analysis of Pepper Fruit Development Provides Insight into Plant Signaling Transduction
Source: Int J Mol Sci. 2020 Mar 13;21(6):1962. doi: 10.3390/ijms21061962 (PMC7139842; doi:10.3390/ijms21061962)

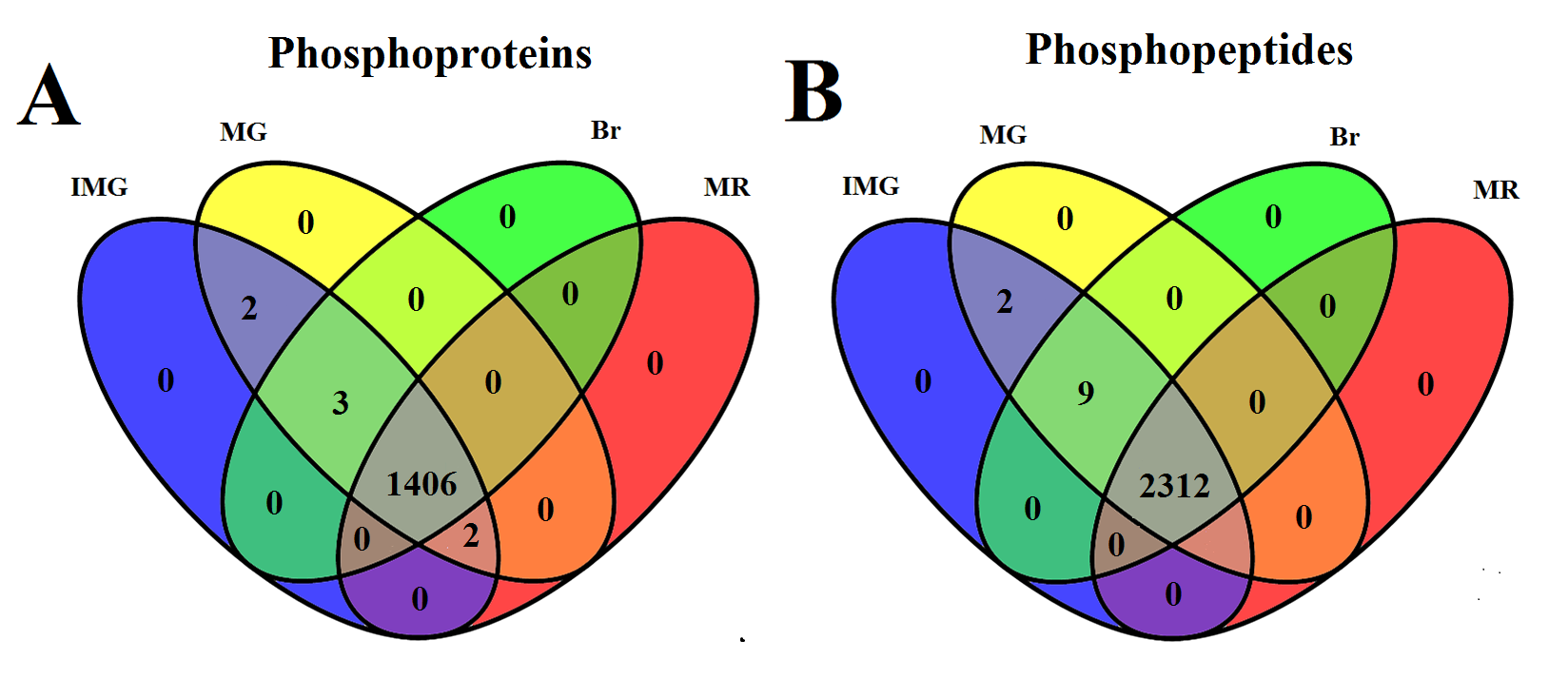

Supplement: Supplementary file 1 [file ijms-21-01962-s001.zip › Supplementary files/Figure S1.tif]
